# Hidden labour: the skilful work of clinical audit data collection and its implications for secondary use of data via integrated health IT

**DOI:** 10.1186/s12913-021-06657-0

**Published:** 2021-07-16

**Authors:** Lynn McVey, Natasha Alvarado, Joanne Greenhalgh, Mai Elshehaly, Chris P. Gale, Julia Lake, Roy A. Ruddle, Dawn Dowding, Mamas Mamas, Richard Feltbower, Rebecca Randell

**Affiliations:** 1 Faculty of Health Studies, University of Bradford, Bradford, UK; 2 Wolfson Centre for Applied Health Research, Bradford, UK; 3 School of Sociology and Social Policy, University of Leeds, Leeds, UK; 4 Faculty of Engineering and Informatics, University of Bradford, Bradford, UK; 5 School of Medicine, University of Leeds, Leeds, UK; 6 Leeds Teaching Hospitals NHS Trust, Beckett Street, Leeds, UK; 7 School of Computing, University of Leeds, Leeds, UK; 8 School of Health Sciences, University of Manchester, Manchester, UK; 9 School of Primary, Community & Social Care, Keele University, Keele, UK

**Keywords:** Clinical audit, Interoperability, Data collection, Data quality, Electronic patient records, Registries

## Abstract

**Background:**

Secondary use of data via integrated health information technology is fundamental to many healthcare policies and processes worldwide. However, repurposing data can be problematic and little research has been undertaken into the everyday practicalities of inter-system data sharing that helps explain why this is so, especially within (as opposed to between) organisations. In response, this article reports one of the most detailed empirical examinations undertaken to date of the work involved in repurposing healthcare data for National Clinical Audits.

**Methods:**

Fifty-four semi-structured, qualitative interviews were carried out with staff in five English National Health Service hospitals about their audit work, including 20 staff involved substantively with audit data collection. In addition, ethnographic observations took place on wards, in ‘back offices’ and meetings (102 h). Findings were analysed thematically and synthesised in narratives.

**Results:**

Although data were available within hospital applications for secondary use in some audit fields, which could, in theory, have been auto-populated, in practice staff regularly negotiated multiple, unintegrated systems to generate audit records. This work was complex and skilful, and involved cross-checking and double data entry, often using paper forms, to assure data quality and inform quality improvements.

**Conclusions:**

If technology is to facilitate the secondary use of healthcare data, the skilled but largely hidden labour of those who collect and recontextualise those data must be recognised. Their detailed understandings of what it takes to produce high quality data in specific contexts should inform the further development of integrated systems within organisations.

## Background

Secondary use of patient data via integrated health information technology (HIT) is fundamental to many policies and processes within healthcare worldwide, including operational and financial practices; audit and quality improvement; and research [1–4]. Apart from enabling data originally collected for one reason (such as clinical care) to be repurposed in multiple ways, it also has potential to maximise efficiency and improve the safety of care [5–8]. Viewed from a broader perspective, such repurposing is integral to societal and scientific trends associated with big data and the operation of predictive analytics on massive datasets to forecast the behaviour of individuals and populations [9–11].

However, moves towards fully integrated HIT across which data can be shared have met with mixed results internationally [12–17]. From a sociological perspective, Berg and Goorman [18] suggest that the profoundly contextual nature of health information is at the root of such difficulties; it is not simply a commodity which can be transported smoothly from one system to another, provided the correct technological connections are in place. According to their ‘law of medical information’: ‘the further information has to be able to circulate (i.e. the more different contexts it has to be usable in) the more work is required to disentangle the information from the context of its production’ (p.52). Edwards [9, 19] uses the metaphor of friction to express the resistance generated when two interfaces interact in this way (whether these exist between machine parts or between systems and people), to which Boyce [20] adds the concept of the ‘second-order friction’ (p.56) that results when data from one system or infrastructure are repurposed in another. In such potentially unruly contexts, Swinglehurst and Greenhalgh [21] draw attention to the importance of the work of those whose role is *do* this repurposing - ‘to make ‘usable’ and ‘useful’ (i.e. to recontextualise) in local sites of practice those technologies which may have been designed at a distance’ (p.2) - and they call for more research into this ‘invisible work’. This research is particularly needed in *intra*-organisational contexts, given that most work to date has focused on *inter*-organisational HIT integration.

Here, we elucidate that hidden, intra-organisational labour, using findings from a study about use of National Clinical Audit (NCA) data for quality improvement in English NHS hospitals. NHS providers in the United Kingdom participate in over 50 NCAs, which disseminate detailed data about patient treatments and outcomes in different clinical specialities or conditions, with a view to minimising variation in the quality of care and promoting improvement. The audits have been described as a national treasure [22], offering a rich source of information for quality assurance, improvement and research, and they have, for example, played a key role in COVID research [23–25]. These achievements have resource implications, and data collection in particular can be resource-intensive [22, 26, 27]. In this paper, the audits are used as an illustration of large-scale data collection with the potential for secondary data use, from the perspective of those involved. Drawing on interviews with, and ethnographic observations of, the clinical and administrative staff responsible for these processes, we seek to understand the nature of this labour more fully, including why so much of it is needed and what constrains further use of integrated data repositories that can share data intra-organisationally for multiple purposes. We conclude by considering the implications of these findings more widely, for other initiatives that seek to promote use of integrated datasets.

## Methods

### Study design

The findings presented here derive from a wider study that explored the use of NCA data for quality improvement within hospitals, to inform the development and evaluation of web-based, interactive NCA quality dashboards [28]. The study was conducted in five phases, incorporating qualitative interviews and ethnographic observations. These captured the fundamental role played by the people who collected, validated and reported NCA data, on which this paper focuses.

### Sample

Our sampling strategy encompassed variation in hospitals, NCAs and user groups, whilst also covering a range of IT systems and processes, to promote the generalisability of our findings. Data were collected across five English NHS hospitals, including three large Teaching Hospitals and two smaller District General Hospitals. Many participants worked with multiple NCAs, but to obtain a more detailed picture of their use, we focused on two audits: the Myocardial Ischaemia National Audit Project or MINAP [29] and the Paediatric Intensive Care Audit Network or PICANet [30], which are delivered by different suppliers, involve different clinical specialities and professional groups, and incorporate both process and outcome measures. All participating hospitals offered cardiology services and contributed data to MINAP, while only the Teaching Hospitals had Paediatric Intensive Care Units (PICUs) and contributed to PICANet: thus, in total, the study involved eight clinical units (five cardiology departments and three PICUs).

Using purposive and snowball methods, in the first phase of the research we interviewed 54 participants working in clinical and non-clinical roles. Twenty of the staff interviewed – 12 of whom were clinicians and eight in non-clinical roles - were involved substantively in collecting or validating data for the audits (i.e. data collection or validation was part of their role): see Table 1. This paper is based largely on these participants’ accounts. Later, we carried out ethnographic observations and informal interviews in the cardiology departments and PICUs (102 h: see Table 2). These observations afforded opportunities to examine the work of audit support staff in action, including the processes, systems and technologies they used for NCA data collection, processing and reporting.

**Table 1:** Interview Participants

Roles	Teaching Hospital 1		Teaching Hospital 2		Teaching Hospital 3		District General Hospital 1		District General Hospital 2		Total (A)	Total (B)
	A	B	A	B	A	B	A	B	A	B		
**Institutional/divisional managers & committee members**	7	1	2	0	3	1	2	1	2	0	**16**	**3**
**Clinicians**	6	4	4	0	8	4	3	1	6	3	**27**	**12**
**Non-clinical support staff in clinical units**	1	1	2	2	3	2	0	0	0	0	**6**	**5**
**Staff in other roles**	3	0	1	0	0	0	1	0	0	0	**5**	**0**
**Total**	**17**	**6**	**9**	**2**	**14**	**7**	**6**	**2**	**8**	**3**	**54**	**20**

A = Total numbers interviewed

B = Number of total involved substantively in NCA data collection or validation

**Table 2:** Ethnographic observations

Types of observation	TH1 hours	TH2 hours	TH3 hours	DGH1 hours	DGH2 hours	Total hours
Ward & ‘back office’ observations, including data collection & validation	25	24.5	17	13.5	2	**82**
Meetings observations & informal interviews	7.5	3	4	2.5	3	**20**
**Total**	**32.5**	**27.5**	**21**	**16**	**5**	**102**

### Data collection and analysis

The initial interviews took place between 30th November 2017 and 6th June 2018, using a schedule developed by the research team, which was reviewed by the study Lay Advisory Group and revised, in light of their feedback, to ensure they covered topics relevant to patients. The interviews were conducted by NA, LM and RR, and ranged from 33 to 89 min, with a median length of 57 min. They included a discussion of participants’ backgrounds and roles, their involvement with and use of NCA data, and the circumstances that supported or constrained such use. Audio-recordings of the interviews were transcribed verbatim and anonymised.

Then, between 21st June 2019 and 13th February 2020, NA and LM carried out ethnographic observations of practices on wards and in offices, where data were collected and validated (82 h). They also engaged in informal interviews with staff to check understandings and explore issues in more depth and observed a range of meetings where NCA and other data were reported (20 h). Detailed field notes were taken on site, and later written up.

Thematic analyses were undertaken of both interview and ethnographic data. Our approach was informed by Framework Analysis [31], developed for use with qualitative data in applied policy research. This method involves familiarising oneself with the data through repeated reading of transcripts, before developing a thematic framework, indexing and then interpreting and synthesising the data in more depth using charts and maps. Our thematic frameworks were developed by the research team and included a framework for the interviews and a separate but complementary framework for the ethnographic observations. The research team agreed initial codes for indexing the data and then indexed five interview transcripts and four sets of ethnographic field notes to test the applicability of codes and assess agreement. Codes were refined and definitions clarified where there was variation, and refined codes were applied to all transcripts, using *NVivo 11*. Next, to facilitate data interpretation and synthesis, we developed narratives that linked cognate themes, enabling us to examine practices within and across cases, and to explore convergence and divergence in participants’ responses. In this paper we draw particularly on a narrative about data collection, which synthesised findings on systems used in the different units to collect, validate and manage data; how data were analysed for reporting; and the challenges experienced in carrying out this work.

### Ethics

The University of Leeds School of Healthcare Research Ethics Committee gave ethical approval for the study (approval number: HREC16–044). For the initial interviews, all participants received an information sheet setting out the study’s aims, how their input would be used, and confidentiality assured, to which they gave their written, informed consent. Where face-to-face interviews could not be arranged and telephone interviews took place instead, verbal consent was recorded.

During the ethnographic observations, we displayed a poster which explained study aims, use of findings and confidentiality. In addition, the researchers provided more detailed information sheets to ward managers and other staff who requested further information. As is customary in ethnographic studies, it was not feasible to obtain written consent from all staff in the vicinity while undertaking observations, and so a written consent form was not used, but the poster, information sheet and the researchers themselves made it clear that staff had no obligation to be observed, and were free to decline before, during and up to 48 h after observation. In the more controlled environment of the meetings observed and informal interviews conducted at this stage, an information sheet was given to participants, and their written, informed consent was obtained.

## Results

During the study, we were struck by the sheer volume and complexity of labour required to collate data for clinical audit. In part, this was due to the amount of data required. NCAs typically require much data from healthcare providers: the MINAP audit, for example, has 130 separate data fields [29]. However, the diverse and distributed nature of the data was also a factor. Although some audit fields may require information that is not already contained within hospital systems (MINAP, for instance, captures detailed process of care data, which do not tend to be represented in standard HIT), they also include more routine information, such as patient demographics and treatments. Such data are commonly captured within different HIT systems including hospital Patient Administration Systems (PAS) and, in those hospitals that have them, Electronic Patient Records (EPR).^1^ In theory, these existing data – at least basic demographic data - could be put to secondary use within NCA records, feeding digitally into those records and thereby removing the need for staff to collect and validate them separately. In practice, however, we found that whilst all clinical units in the study made some use of such routinely-collected data in their NCA returns (even if only for cross-referencing purposes), the population of shared fields was not straightforward (see Table 3 below). Rather, staff spent much time gathering and checking data from a range of sources, often copying information from digital systems to paper forms, before rekeying it into local databases or NCA web portals, and we observed variations across sites in how this was achieved. We explore this complex work below, referring to key individuals involved using pseudonyms, to protect their anonymity.

**Table 3:** Summary of NCA data completion approaches in the study sites

Hospital	Service	Data collection processes	Extent to which shared data are used
Teaching hospital 1 (TH1)	Cardiology	‘Jim’, a cardiology nurse specialist, works full-time on NCA data returns. He completes a hard-copy data form for each eligible patient, using information from paper medical notes and the Trust’s HIT, and keys the data into a departmental database. The data are then uploaded by the hospital’s IT department to MINAP.	MINAP and PICANet data returns are separately generated rather than being auto-populated from within hospital HIT systems. NCA data are, however, checked or cross-referenced against these systems: for example, to ensure that all eligible patients are included within the audit return, and that dates/interventions within hospital HIT match those separately generated for the NCA.
	PICU	‘Anne’, a full-time non-clinical audit co-ordinator, collates data collected by nurses and registrars on the ward on paper data collection forms, provided by the audit supplier. She checks this information by comparing it with the ward admissions book, handover sheets, patient notes, the PAS and EPR, and then keys the data into a local Access database, before uploading the data to PICANet’s web portal. She also enters some additional data, not stored in her database, separately into the portal.	
Teaching hospital 2 (TH2)	Cardiology	‘Neil’, a full-time, non-clinical member of staff, is responsible for several NCA data returns. His attempts to involve staff on the wards in MINAP data collection via a paper data collection form have met with limited success, and he tends to collect the data himself without recourse to a form, storing them in an Access database before uploading to the supplier. He obtains some information in bulk by querying the hospital’s data warehouse, exporting it to an Excel spreadsheet and then importing it into his Access database. He also obtains information from paper patient notes and digitally-stored discharge letters and ambulance systems.	The MINAP data return is separately generated rather than being auto-populated from within hospital HIT systems, although Neil minimises re-keying of data by importing data from other systems where possible.
	PICU	‘Grace’, a part-time audit clerk, is responsible for the PICANet return for this small PICU. She does not have a dedicated database or spreadsheet, but transfers data direct into PICANet’s web portal from four or five different systems, including the Trust’s PAS and EPR; an electronic system that contains appointments and transport data; and paper patient notes.	The PICANet data return is separately generated rather than being auto-populated from within hospital HIT systems, although data are copied between systems whenever possible, rather than re-keyed.
Teaching hospital 3 (TH3)	Cardiology	MINAP data are stored in an in-house database. NSTEMI data (Non-ST-elevation myocardial infarction: a milder type of heart attack) are collected via a paper data collection sheet by two cardiology nurse specialists, ‘Molly’ and ‘Louise’, alongside their clinical duties, when they see patients. For patients they don’t see themselves, they obtain data from a range of sources, including the PAS and EPR, ambulance service records and paper notes. STEMI data (ST-elevation myocardial infarction: a serious type of heart attack) are collected by a full-time non-clinical assistant, ‘Amy’, who inputs them directly into the database from sources such as paper notes; an electronic system that stores patient letters; and bulk reports of patients’ blood results. When MINAP data are complete, the department’s IT team runs a report and uploads the data to the supplier.	MINAP and PICANet data returns are separately generated rather than being auto-populated from within hospital HIT systems, although data are checked or cross-referenced against these systems.
	PICU	PICANet data are recorded by nurses and doctors on paper data collection forms, after which they are input to a specially designed Excel spreadsheet and direct to PICANet’s web portal. This was done by a full-time database manager, ‘Adam’, until his recent departure to a new job, and is now done by another non-clinical staff member, ‘Sara’, supported by a research nurse.	
District General Hospital 1 (DGH 1)	Cardiology	Data are collected directly from paper patient notes by cardiology nurses, who enter them to a departmental database. The return is co-ordinated and uploaded to the supplier by ‘Sue’, a nursing team leader, who runs reports to check and clean the data and uploads them to MINAP. Sue took on the role recently from another experienced nurse.	In both hospitals, the MINAP data return is separately generated rather than being auto-populated from within hospital HIT systems, although data are cross-referenced.
District General Hospital 2 (DGH 2)	Cardiology	‘Linda’, an experienced cardiac assessment nurse, works with two other nurses on MINAP data collection alongside clinical duties. Where eligible patients are admitted directly to the Coronary Care Unit, nurses there begin completing paper data collection forms, which are collected daily by the MINAP team, who add discharge data and then key the results direct to the MINAP web portal (there is no in-house database). For other patients, MINAP team members complete the forms themselves, consulting a range of different systems, such as the PAS and paper notes.	

### ‘Grinding it out’: collecting data from multiple systems in resource-limited contexts

In the hospitals in our study the data needed for NCA records were not held in single electronic systems, but in multiple locations. There was much use of paper-based records, especially patient notes, but even where sources were electronic, they were not always linked with each other. Gathering data from different, unintegrated systems was time-consuming and arduous: hard work, which ‘Molly’, a cardiology nurse involved in MINAP data collection at Teaching Hospital 3 (TH3) described as: ‘*we have to grind it out’*.

TH2 appeared to have the most automated systems for NCA data collection, and participants there re-entered data into digital systems less than in the other hospitals (see Table 3). In cardiology, for example, ‘Neil’, a data analyst with advanced IT skills, identified the information he needed from his hospital’s data warehouse (a large data repository designed to facilitate data analysis) by submitting Structured Query Language requests to the warehouse. In this way he was able to derive bulk data reports for export via an Excel spreadsheet into the Access database he used to store MINAP data. Yet even here the process was partly manual: though he hoped to move towards further automation, at the time of our observations Neil entered queries himself each time he updated the MINAP record. Moreover, he was unable to obtain all MINAP data in this way and needed to refer too to separate ambulance systems and digitally stored discharge letters, as well as to paper case notes. Neil carried out this work amongst many other responsibilities and estimated that it took him between 30 and 60 min to collect MINAP data for each patient, of which there were around 800–1200 a year.

Similarly, ‘Grace’, a part-time audit clerk in the TH2 PICU, used four or five different systems to populate the PICANet record, copying and pasting data from the former to the latter: a repetitive process that, whilst minimising re-keying, she regarded as old-fashioned. Grace would have preferred HIT to feed the PICANet record automatically, but the requisite technology was not available, and she had to transfer the data manually: a job that, far from being a straightforward ‘cut and paste’ matter, required skill and discretion, as the following extract from our field notes shows:

*[Grace] opens the PAS and PICANet Web [*the audit supplier’s web portal*] and displays them in two side-by-side windows on her monitor. She gets information about patients from the PAS, copies the data and pastes it into PICANet Web. […] She checks other systems too, including a system that contains patient flow data, appointments, and the transport round. [Grace] uses it because ambulance staff record PIMS* [Paediatric Index of Mortality] *data on it when they take patients’ blood gas levels, and she compares the patient flow system with PAS to check she’s got the right PIMS information, reading nursing notes when she finds an anomaly in the data. (Observation of Grace, extract from field notes, TH2).*

In other hospitals in the study, data collection was less automated. In District General Hospital 2 (DGH2), for example, nurses used paper forms to complete the MINAP return, drawing from a range of electronic and paper records, including the PAS and patient notes. Having no local system in which to store data from these forms, they were entered directly to the MINAP web portal. ‘Linda’, an experienced cardiology nurse who co-ordinated MINAP work, emphasised the labour involved in accessing these different, unintegrated systems, which she and her colleagues undertook alongside many clinical duties. She called for further automated data sharing to reduce the workload:

*There are so many separate ways of collecting the data […] So when the patient has gone home, if we haven’t managed to get those notes […] we have to open the ICE* [Integrated Clinical Environment: a widely used pathology system] *letter, we have to find the blood results, we have to see if they’ve had an echo* [echocardiogram: an ultrasound scan of the heart] *– and these are all separate systems that we’re all looking in – and it’s extremely time-consuming, whereas if these systems could talk to one another, a lot of the stuff could already be filled in, or it’s down to IT, whether they can afford to do it, whether they can afford the maintenance of it, and things like that. But I do think that that is not beyond the realms of possibility. (Linda, Phase 1 interview, DGH2).*

Linda had asked her hospital’s busy IT department if they could introduce such a system, but this had not yet been possible and she commented, pragmatically: ‘*Like the usual hospital things, it takes two or three years to sink through’*.

Difficulties in providing systems that could, as Linda put it, ‘talk to one another’ appeared, in part at least, to be linked to resource limitations in the hospitals in the study. These limitations were reflected in the dated technology used by some staff in clinical units. Neil’s Access database for MINAP in TH2, for example, was around 14 years old. Anne’s database in the TH1 PICU was of a similar vintage, having been developed by a junior doctor on rotation there; no-one since had had the skills or time to update it, so as PICANet added or revised fields in subsequent years, Anne had to collect those data separately and input them to PICANet’s web portal manually. The TH2 PICU and DGH2 cardiology department had no dedicated digital storage for NCA data at all and had to input directly to the supplier websites. Other units, however, had access to more up-to-date hardware and software, and some - for example, the TH1 and DGH1 cardiology departments - used databases designed by third-party suppliers.

### Double data entry and use of paper data collection forms

Several clinical units in the study used paper forms to collect NCA data. Although this involved writing and then re-keying information also held within digital systems, paper forms were used because staff believed they had practical advantages in terms of their flexibility and portability. Given the multiplicity of systems from which NCA data were derived, forms provided a single location where all data could be gathered, acting as manual data warehouses, as it were. They also made it easier to distribute and contemporise the work of data collection. A form could, for example, be added to the paperwork that clinicians must complete during patient care, and some of the forms served multiple uses. In TH3, for example, Molly and her nursing colleague ‘Louise’ explained that their MINAP form was not only a data collection tool, but also had clinical utility:

*Louise: It ties in with what you want to know about the patient, and it also ties in with MINAP, and it also ties in with what clinically you need to know, like the patient’s risk factors.*

*Molly: And we can utilise it. We have other sort of projects going along that we’re trying to get patients through to the lab within a certain amount of time, and we can use what we collect on that sheet for that as well. (Molly & Louise, Phase 1 interview, TH3).*

Importantly, forms could be completed by different clinicians when patients (and their paper notes) were present on the wards, making it easier to check any anomalies that arose. Systems that at first sight appeared more efficient did not have these advantages. For example, although Neil in TH2 partly populated his Access database with data from his hospital’s data warehouse, the entire burden of MINAP data collection also fell on him and had to be done retrospectively. For this reason, Neil had tried to introduce a paper form to be completed by clinicians, even though this would have involved him in subsequent re-keying, but take-up was limited at the time of our observations owing to staff shortages and the pressure of other work on Neil and clinical colleagues.

### Lacking trust in shared data quality

Another reason several units used paper data collection forms to gather NCA data was that staff did not always trust the quality of data in their hospitals’ digital systems, and therefore did not want simply to import ‘raw’ routinely-collected data into their carefully curated and validated NCA records, even were this option available to them. ‘Jim’, a nurse responsible for the MINAP return in TH1 put it like this:

*I don't trust the data that goes onto PAS […]. With PAS, you're supposed to put them on within, I think, half an hour of admission to the ward, but the wards are that busy, they just can't do it, it's just impossible. […]. So yeah, I collect all that data. (Jim, Phase 1 interview, TH1).*

In these units, data recorded on forms were then checked against the PAS and other electronic or paper systems before being input to local databases and/or NCA supplier websites, providing opportunities for anomalies to be addressed through triangulation and discussion with colleagues. ‘Anne’, a non-clinical audit co-ordinator in the TH1 PICU, and her clinical colleagues, highlighted the importance of this process. In the past, their unit had been flagged with an outlying standardised mortality ratio by PICANet, which – following many hours of intensive research by Anne and the unit’s clinical lead for the audit, a consultant paediatrician - turned out to be caused by inaccurate data rather than clinical issues. One reason for the inaccuracy, they discovered, was the involvement in data collection of different individuals and teams with different understandings about how the data would be used. As a result, staff were subsequently strongly motivated to maintain high quality data, comprehensively checked by Anne and the clinical lead for the audit, and involving as few other people or shared data as possible; indeed, clinicians in the PICU now regarded their PICANet data as a ‘gold standard’, far more accurate than data in other Trust-wide HIT:

*The PICANet data, via Anne, to me is the gold standard of our activity. […] I know what Anne does and I know that her level of form completion is very good and, therefore, I can rely on the data I get from that. Whereas there are too many variables in the other data collection for me to sort of have total faith in. (Head of PICU, Phase 1 interview, TH1).*

Given this background, staff were wary of moves within the Trust towards further automatic data sharing and feared that the replacement of their local PICANet Access database by digitally generated data from the EPR and other data platforms would reduce data quality. Staff in the TH3 cardiology department and PICU reported similar views, giving examples of inaccurate data caused by many hands being involved in data collection, which led to problems such as accurate data from one system being overwritten by less accurate data from another during bulk data imports.

### Skilful labour

The work of collecting and inputting data for NCAs required skill and judgement, and several of those involved, whether clinicians or non-clinical staff, had built up expertise over many years. In DGH2, for example, Linda, a cardiac assessment nurse, had worked with MINAP data for 19 years, whilst Anne, the non-clinical audit co-ordinator in the TH1 PICU, had 16 years’ experience with PICANet. Given the expertise required to do this work, there were differences of opinion about whether non-clinical staff should be involved. Molly, a cardiology nurse specialist who had co-ordinated the MINAP return in TH3 for around 15 years, expressed doubts about the accuracy of data collected by non-clinical staff in other hospitals:

*So my concern has always been with other Trusts, when it’s not clinical people who are involved in MINAP, because I just don’t think it’s accurate enough if you’ve not got clinical people doing it, because you need to be able to read ECGs* [electrocardiogram: a test to check the heart’s rhythm and electrical activity] *to know whether an ECG was diagnostic or not. You can’t just put the time of any ECG. It’s got to be the one that was diagnostic. (Molly, Phase 1 interview, TH3).*

Here, we see that clinical knowledge is needed to understand the context of data production and choose which data are required for the audit. In line with this, several non-clinical staff members had developed a knowledge of clinical processes well beyond what might be expected in their roles, so that they could make such decisions. Anne in TH1, for example, had received training to understand clinical terminology, whilst in TH2, Neil, although not a clinician, used his scientific background and 15 years’ experience in the role to interpret ECG charts. Like the other non-clinical staff in the study, both Anne and Neil consulted clinicians when they were unsure. ‘Adam’, a non-clinical database manager in the TH3 PICU, believed this skilful work relieved the administrative burden on clinicians:

*So when I started I was just taking the PICANet forms, putting them onto PICANet, really basic kind of data input stuff. And then as it went on, it was getting more involved in understanding why we’re collecting that data, then trying to educate other staff into why. And then cross-referencing the PICANet forms against our electronic system […], trying to fill in the blanks because the problem is with the PICANet forms, they don’t always get filled in. ‘Cause nurses feel like they’re duplicating or triplicating work at times. You know what it’s like. They’re nursing two-to-one on the patients, they just literally don’t have time to fill in the paper forms. So [my work] has evolved into more understanding the daily interventions and things like that, and then obviously the role has then developed into bespoke data requests for the units, things like bed occupancy, elective, cancelled operations. (Adam, Phase 1 interview, TH3).*

Adam pointed out that his expertise enabled him to respond to ‘bespoke data requests’, by feeding PICANet data into reports on outcomes such as bed occupancy, and this was the case in other units in the study too, where staff in clinical units provided reports of NCA data to inform quality assurance and improvement activities. Molly, for example, used MINAP data in monthly governance meetings to identify and address delays in treatment, and pointed to the significance of data collection in that work:

*Having to input all that data makes you realise: why has that not been done? That patient’s had this diagnosis but they’ve not had an echo requested, and why not? So until you have somebody that goes along and puts that all in, you might never realise actually they should have had that done or that done, and it wasn’t requested. […] It allows you to pick that up, […] it’s just very time-consuming. (Molly, Phase 1 interview, TH3).*

In other words, according to Molly, involvement in the minutiae of data collection, whilst time-consuming, highlighted areas of concern that required clinical attention: a key stage in quality improvement that would need to be addressed differently were data collection to be entirely automated.

## Discussion

This article reports, to the best of our knowledge, one of the most detailed empirical examinations undertaken to date of the practices involved in repurposing healthcare data for NCAs. We observed clinical and non-clinical staff generating NCA records through painstaking, skilful, ‘behind-the-scenes’ work. Some data required by the NCAs already existed in other HIT systems in the hospitals and were available, in theory, for secondary use in the audit records. However, although staff in some units copied or downloaded data directly into those records from hospital-wide digital systems, the population of shared fields was not automatic or even always digital, as envisaged, for example, in strategies that promote interoperable systems which can exchange meaningful data digitally [5, 8]. Instead, double data entry and use of paper data collection forms were common practices.

Participants’ continued use of manual technologies and the duplication of work this entailed did not spring from a lack of IT skills or an antiquated clinging to paper, however; indeed, many were keen to move towards further automation. Rather, they were skilful pragmatists, who recognised and utilised the flexibility and portability afforded to them by paper-based approaches to data collection. They worked as they did for good reasons, then, such as safeguarding data quality or assuring and improving service quality, and their largely hidden work played an important role in developing end-user trust in the data.

Trust was a key driver in data use. Bonde and Bossen [32] found similar links of trust and cooperation between data workers and clinicians when studying the development of quality and patient-value indicators in Danish hospitals. They highlight the importance, in this, of shared experience and iterative dialogue between both groups, facilitated when they worked together in the same department and hampered when data work was later centralised. Likewise, in our study, audit support staff were based in the clinical units for which they collected data (and, in several cases, had been there for many years), and were able to engage in local discussions with colleagues if queries arose. This helped them to develop a deep understanding of the data, which was critical in building and maintaining trust in its quality, and its consequent use for quality assurance and improvement.

As Dixon-Woods et al. [26] point out, such data work, far from being ‘an abject form of labour’ (p.8), is undertaken as a ‘professional duty’ (Ibid.), drawing on discrimination and expertise. Berg and Goorman [18] relate the skill required to undertake such work with the complexity of disentangling healthcare data from one context to fit another, a finding echoed in several other studies [3, 4, 17, 26, 33–35]. We, too, witnessed this skill, when, for example, watching Grace in TH2 cross-reference several systems to ensure she reported accurate Paediatric Index of Mortality data to PICANet. Edwards’ [9, 19] metaphor of data friction reflects the ‘grind’, as our participant ‘Molly’ put it, of this skilful, hard work, whilst Bonde and Bossen [32] draw attention, too, to the *generative* implications of friction – the sparks it can ignite - like the opportunities for quality improvement that Molly was prompted to identify when ‘grinding out’ MINAP data. Returning to the depletive impacts of friction, Edwards [9] notes that two processes act to reduce it: precision – in this context, the precision of highly accurate systems that fit together smoothly - and lubrication. Lubrication eases the interaction between systems, even when interfaces are imperfect, and Edwards likens it to the facilitative operation of ‘ephemeral, incomplete, *ad hoc*’ (p.684) communicative processes between those who share data, to keep things running.

Sociologically-informed and feminist accounts of the creation and recreation of healthcare records paint a similar picture, in which meaningful data emerge from the complex, untidy, heterogeneously-motivated interactions of people and digital programs within socio-cultural, institutional and political systems [13, 21, 36, 37]. From this angle, Swinglehurst and Greenhalgh [21] reframe the ‘invisible work’ (p.2) of data collection as knowledge work, which involves ‘an interweaving of tedious activity, mindful judgment and practical reasoning’ (p.2), noting that:

*Current interest in large datasets and the potential for health data to be put to an ever widening array of secondary uses tends to obscure the socially complex work that lies in the details of how data gets onto the record, and we suggest that this presents an important, often overlooked agenda for research on the quality of health care* [21]*.*

Our study seeks to add to this overlooked agenda, by highlighting such work and calling for its positive and generative effects to be maintained in future, more digitally integrated healthcare systems. We suggest two factors that can facilitate intra-organisational, secondary use of patient data. First, the data that feed such systems must be accurate to avoid the problem of ‘garbage in - garbage out’ [38] or, to prevent this, the time-consuming cross-checking and duplication identified by our participants. Second, software and data exchange interfaces between linked systems must be appropriately defined, both technically and semantically, and the complexity of the links between them navigated effectively. Crucially, both factors need input not only from IT specialists, but also from the people who understand the data and their contexts, meanings, dependencies, provenances, quality and limitations: trusted people such as the clinical and non-clinical audit support staff whose work is highlighted here. These individuals can make significant contributions to the design and development of integrated systems within organisations.

Our findings also point to the difficulties in realising *fully* interoperable health information systems, and the possibility that they may never incorporate wholly the responsiveness and informed discretion that human actors bring: qualities that Winthereik and Vikkelsø [39] characterise as ‘interpretative flexibility’ (p.61). Those authors call for systems to be designed in ways that enable the staff who exercise this flexibility to continue to span the boundary between messy reality and standardised requirements. With this in mind, we suggest that designers of integrated HIT aim to strike a balance between automating the most labour-intensive parts of data integration, whilst designing interfaces that empower users to assess integration outcomes and, where necessary - for example, if data quality issues arise – to continue to use their own skill and ingenuity to address problems. In Edwards’ [9] terms, such systems are as precise as possible, but are also open to lubricative processes to keep running.

### Strengths and limitations

By studying two large, well-established audits, MINAP and PICANet, in distinct clinical fields, used by staff in different hospitals with diverse HIT systems, we were able to capture much variation, which promotes the generalisability of our findings to a degree. We have reflected on the complexity of this variation and its implications in this paper. However, we do not claim to have represented the full range of audits or levels of digitisation in hospitals, for some of which data collection may be more automated or may differ in other ways. For example, we saw some evidence that clinicians were motivated to maintain up-to-date, accurate data for audits which reported on their performance as individual operators, like the audits of the British Association of Urological Surgeons (BAUS), which could reduce the need for validation and cross-checking of these data. Future research might usefully explore data collection in these types of audit more extensively than we were able to.

Further, the sample relevant to the focus of this paper – staff involved in NCA data collection and validation - was small, with only 20 participants working substantively in this area. This enabled us to explore their work in detail in qualitative interviews and ethnographic observations but limits the generalisability of our findings. We had hoped to spend more time observing staff, but the COVID-19 pandemic cut short our endeavours, reminding us of the contingent and unpredictable nature of data collection, whatever the context, and the need for pragmatic responses. We point therefore to the emergent and situated nature of our findings and present them tentatively, as a contribution to the wider debate on the use of integrated datasets.

## Conclusion

Secondary use of patient data via integrated HIT has been linked with advances in data accessibility and quality, enhanced patient safety and workforce efficiency [5–8]. If these developments are to be realised more fully, the skilled but largely hidden labour of the people who collect and recontextualise the data for such uses must be recognised. Their detailed understandings of what it takes to produce high quality data that can be used to assure and improve care quality in specific contexts should inform the further development of integrated systems within healthcare organisations.

## Data Availability

In accordance with the ethical approval for this research, data will be kept until June 2030 and can be accessed by other researchers during this time, subject to the necessary ethical approvals being obtained. Requests for access to this data should be addressed to the corresponding author.

## Abbreviations

DGH: District General Hospital
ECG: Electrocardiogram
EPR: Electronic Patient Records
HIT: Health Information Technology
ICE: Integrated Clinical Environment
MINAP: Myocardial Ischaemia National Audit Project
NCA: National Clinical Audit
NHS: National Health Service
NSTEMI: Non-ST-elevation myocardial infarction
PAS: Patient Administration System
PICANet: Paediatric Intensive Care Audit Network
PICU: Paediatric Intensive Care Unit
PIMS: Paediatric Index of Mortality
STEMI: ST-elevation myocardial infarction
TH: Teaching Hospital

## Footnotes

1 At the time of our research the three large Teaching Hospitals in the study had both PAS and EPR systems, alongside many other (not always integrated) HIT applications. The two smaller District General Hospitals had PAS and other HIT, but did not yet have EPRs, although they planned to introduce such systems.
